# Characteristics of home oxygen therapy for preterm infants with bronchopulmonary dysplasia in China: results of a multicenter cohort study

**DOI:** 10.1007/s12519-022-00591-9

**Published:** 2022-08-11

**Authors:** Wen-Xing Jiang, Yan-Chen Wang, Hong-Xia Song, Mi Xiao, Fan He, Si-Yuan Jiang, Xin-Yue Gu, Jian-Hua Sun, Yun Cao, Wen-Hao Zhou, Shoo Kim Lee, Li-Ping Chen, Li-Yuan Hu, Chinese Neonatal Network, Shoo K. Lee, Chao Chen, Li-Zhong Du, Wen-Hao Zhou, Yun Cao, Fa-Lin Xu, Xiu-Ying Tian, Hua-Yan Zhang, Yong Ji, Zhan-Kui Li, Jing-Yun Shi, Xin-Dong Xue, Chuan-Zhong Yang, Dong-Mei Chen, San-Nan Wang, Ling Liu, Xi-Rong Gao, Hui Wu, Chang-Yi Yang, Shu-Ping Han, Ruo-Bing Shan, Hong Jiang, Gang Qiu, Qiu-Fen Wei, Rui Cheng, Wen-Qing Kang, Ming-Xia Li, Yi-Heng Dai, Li-Li Wang, Jiang-Qin Liu, Zhen-Lang Lin, Yuan Shi, Xiu-Yong Cheng, Jia-Hua Pan, Qin Zhang, Xing Feng, Qin Zhou, Long Li, Ping-Yang Chen, Xiao-Ying Li, Ling Yang, De-Yi Zhuang, Yong-Jun Zhang, Jian-Hua Sun, Jin-Xing Feng, Li Li, Xin-Zhu Lin, Yin-Ping Qiu, Kun Liang, Li Ma, Li-Ping Chen, Li-Yan Zhang, Hong-Xia Song, Zhao-Qing Yin, Ming-Yan Hei, Hui-Wen Huang, Jie Yang, Dong Li, Guo-Fang Ding, Ji-Mei Wang, Qian-Shen Zhang, Xiao-Lu Ma, Joseph Y. Ting

**Affiliations:** 1 Division of Neonatology, Jiangxi Provincial Children’s Hospital, 1666 Diezihu Avenue, Honggutan New Area, Nanchang, 330038 China; 2 NHC Key Laboratory of Neonatal Diseases, Fudan University, Children’s Hospital of Fudan University, Shanghai, 201102 China; 3 Division of Neonatology, The First Affiliated Hospital of Xi’an Jiaotong University, Xi’an, 710061 China; 4 Division of Neonatology, Children’s Hospital of Fudan University, 399 Wanyuan Road, Minhang District, Shanghai, 201102 China; 5 Division of Neonatology, Shanghai Children’s Medical Center, Shanghai Jiao Tong University School of Medicine, Shanghai, 200127 China; 6 Maternal-Infants Care Research Centre and Department of Pediatrics, Mount Sinai Hospital, Toronto, ON M5G 1X5 Canada; 7 University of Toronto, Toronto, ON M5T 3M7 Canada

**Keywords:** Bronchopulmonary dysplasia, Chinese Neonatal Network, Gross domestic product, Home oxygen therapy, Preterm infants

## Abstract

**Background:**

Home oxygen therapy (HOT) is indicated upon discharge in some preterm infants with severe bronchopulmonary dysplasia (BPD). There is a lack of evidence-based consensus on the indication for HOT among these infants. Because wide variation in the institutional use of HOT exists, little is known about the role of regional social-economic level in the wide variation of HOT.

**Methods:**

This was a secondary analysis of Chinese Neonatal Network (CHNN) data from January 1, 2019 to December 31, 2019. Infants at gestational ages < 32 weeks, with a birth weight < 1500 g, and with moderate or severe BPD who survived to discharge from tertiary hospitals located in 25 provinces were included in this study. Infants with major congenital anomalies and those who were discharged against medical advice were excluded.

**Results:**

Of 1768 preterm infants with BPD, 474 infants (26.8%) were discharged to home with oxygen. The proportion of HOT use in participating member hospitals varied from 0 to 89%, with five of 52 hospitals’ observing proportions of HOT use that were significantly greater than expected, with 14 hospitals with observing proportions significantly less than expected, and with 33 hospitals with appropriate proportions. We noted a negative correlation between different performance groups of HOT and median GDP per capita (*P* = 0.04).

**Conclusions:**

The use of HOT varied across China and was negatively correlated with the levels of provincial economic levels. A local HOT guideline is needed to address the wide variation in HOT use with respect to different regional economic levels in countries like China.

**Supplementary Information:**

The online version contains supplementary material available at 10.1007/s12519-022-00591-9.

## Introduction

Bronchopulmonary dysplasia (BPD) is a common complication of preterm infants, and occurs in as many as 74% of infants born at < 28 weeks of gestation and 11.4% of infants born between 28 and 31 weeks in China [1–4]. BPD is the major cause of prolonged hospital stay for preterm infants and correlates with higher mortality and morbidity [2–5].

Home oxygen therapy (HOT) is recommended for infants with BPD who are stable on low-flow nasal cannula oxygen [6, 7]. HOT accounts for 13.2% of infants with BPD in South Korea [8], 17.2% in New Zealand, 19.3% in Australia [9], 40.5% in the United Kingdom [10], and 65.2% in the United States [11], depending upon the incidence and practice of managing infants with severe BPD in the individual countries. HOT enables early discharge from the hospital, decreases the substantial burden on health care systems, improves patient quality of life, and is not associated with readmission for infants with BPD in regional neonatal intensive care units [12–14].

No consensus or evidence-based practice has thus far been achieved regarding the entry criteria and mode of HOT for infants with severe BPD. Wide variation in the institutional use of HOT exists, ranging from 7% to 95% of infants with BPD in California [12] and 0% to 36.4% in South Korea [8]. A better understanding of the variation in home oxygen use will be important for patient counseling, designing interventions, creating policy, and evaluating the impact on long-term respiratory and other health outcomes [15]; however, such data are lacking in China, and little is known about the role of regional social-economic level in the wide variation of HOT.

Large variations of neonatal care practice exist in different regions in China and lead to significant differences in neonatal morbidity [16]. In addition, regional disparities in infant mortality rate in rural China [17] and in social-economic level might be related to institutional variation of HOT use in China. We applied provincial gross domestic product (GDP) per capita to reflect the economic level in each province.

The objective of this study was to characterize the use of HOT in preterm infants with BPD in China based on the Chinese Neonatal Network (CHNN) data, to explore the role of GDP per capita in the regional variation of HOT use, and to generate information that is beneficial to the design of interventions and the formulation of appropriate policy.

## Methods

### Study design

The CHNN was established in 2018 to implement high-quality, cooperative research and to improve the quality of neonatal care in China. Since January 1st of 2019, the CHNN has collected detailed clinical data from participating hospitals regarding all premature infants with a birth weight (BW) of less than 1500 g or a gestational age (GA) of less than 32 weeks. A standardized, perinatal/neonatal clinical database has thus been generated that is regularly maintained and controlled with respect to quality by the CHNN coordinating center. In 2019, a total of 57 hospitals from 25 provinces in China joined the CHNN, caring for approximately 5% of all very preterm infants (VPIs) in China [18].

This study was approved by the Ethics Review Committee of the Children's Hospital of Fudan University and by all of the participating member hospitals of the CHNN. We herein followed the Strengthening the Reporting of Observational Studies in Epidemiology (STROBE) reporting guidelines for cohort studies.

### Study population

**Fig.1:**
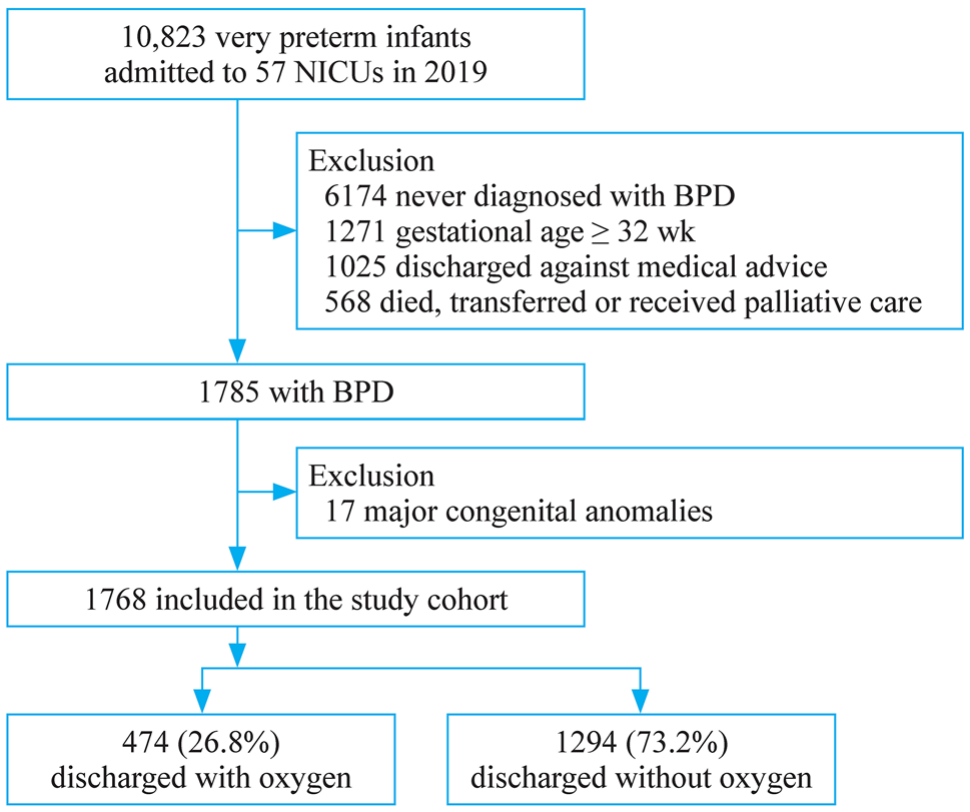
Flow diagram for the study

This was a hospital-based cohort, with all VPIs admitted to CHNN hospitals between January 1, 2019 and December 31, 2019 and assessed for eligibility. Those infants who survived to discharge and were diagnosed with moderate or severe BPD based on 2001 NIH consensus definition were included in our analysis [1]. Infants with major congenital anomalies and those who were discharged against medical advice [18] (meaning that parents terminated treatment before the treating physicians recommended discharge) were excluded. We tracked readmissions and transfers between participating hospitals as data from the same infants (Fig. 1).

### Data collection

Trained data abstractors were responsible for data collection at each hospital, and site investigators were responsible for data quality control for each hospital. The identities of the patients were kept confidential. Data were directly entered into a custom database that possessed built-in error checking and a standard operations and definition manual [19]. The data then were transmitted electronically to the CHNN coordination center of the Children's Hospital of Fudan University. Periodic feedback of erroneous data, data correction, and auditing were performed for each site to ensure the accuracy and consistency of data abstracted from medical records.

### Outcomes

The outcomes included the proportion and characteristics of HOT required for preterm infants with BPD. HOT included any type of respiratory support after discharge, from low-flow oxygen and high-flow oxygen to non-invasive ventilation and invasive ventilation via tracheostomy.

### Definitions

BPD was defined as infants diagnosed with moderate or severe BPD based on 2001 NIH consensus definition, and infants with moderate or severe BPD were included in the analysis [1]. Gestational age was determined using the hierarchy of best obstetric estimate based on prenatal ultrasonography, menstrual history, obstetric examination, or all three. If the obstetric estimate was not available or was different from the postnatal estimate of gestation by more than two weeks, the gestational age was estimated using the Ballard Score [20]. Small for gestational age (SGA), appropriate for gestational age (AGA), and large for gestational age (LGA) were defined according to the Chinese neonatal birth weight values [21]. Sepsis was designated as positive for bacteria in blood or cerebrospinal fluid culture [22]. Breast milk was described as donor breast milk or mother’s own breast milk. Weight gain velocity was defined by the equation: weight change (g/kg/day) = (discharge weight − birth weight)/([discharge weight + birth weight]/2/1000)/length of stay in days from birth to discharge [23]. Necrotizing enterocolitis (NEC) was defined as NEC of stage 2 and above according to the Bell stage [24]. Invasive ventilation included conventional ventilation and high-frequency oscillatory ventilation (HFOV). Non-invasive ventilation included non-invasive positive-pressure ventilation (NIPPV), continuous positive-airway pressure (CPAP), and non-invasive high-frequency oscillatory ventilation (nHFOV). When the infant received more than one mode of respiratory support on the same day, we characterized the respiratory support on that day as the higher level of respiratory support. From lower to higher levels, the respiratory support modes were depicted as non-invasive and invasive, respectively. To determine the influence of provincial economic level on HOT use, provincial gross domestic product (GDP) per capita was applied to determine the provincial economic level, which was abstracted from National Bureau of Statistics of China [25].

### Statistical analysis

Baseline characteristics were initially summarized descriptively. A χ2 test was applied to compare the binary-valued maternal and infant information between infants with and without HOT. For highly-skewed variables, we conducted Wilcoxon test to assess the difference in duration of respiratory support in the NICU between the two groups, using medians and quartiles.

To identify risk factors for HOT use among neonates with BPD, we employed multivariable logistic regression, using a generalized estimating equation (GEE) approach. This approach was used to estimate the model parameters while accounting for cluster effects within each site. Potential risk factors were determined using a previous study and our overall knowledge base—including GA, use of antenatal steroids, percentile of birth weight, length of NICU stay, duration of breastmilk use per 100 NICU stays, doses of surfactant, PDA ligation, NO use, NEC, PMA at discharge, duration of invasive ventilation, duration of non-invasive ventilation, and duration of oxygen use [12].

A funnel plot is widely used for institutional comparisons in which an estimate of an underlying quantity is plotted against an interpretable measure of its precision, so that the control limits form a “funnel” around the target outcome [26]. Variation in HOT use was also summarized within site levels of the CHNN by calculating the observed percentages of HOT use. Expected proportions of HOT use by hospitals were estimated based on the aforementioned model. We then calculated an indirectly standardized ratio using the equation: observed proportion of HOT/expected proportion ratio, wherein we excluded hospitals with < 5 BPD cases to ensure a stable estimate for comparison (four sites). Funnel plots were used to assess the site performance of HOT, which followed a Poisson distribution [26]. Based on the funnel plots, 52 sites were divided into three groups: over-performance (above the + 95% control limit), normal performance (within control limits), and underperformance (below the − 95% control limit). Provincial GDP per capita abstracted from the National Bureau of Statistics of China was compared among the three performance groups with the Kruskal–Wallis test [25].

To determine the impact of economic development in Chinese cities on HOT, the provincial expected proportions of HOT were estimated based on the above-mentioned model with a calculation of the indirectly standardized ratio. Thus, we exploited linear regression to determine a trend in the indirectly standardized ratio with increasing GDP per capita. In addition, as a sensitivity analysis, we applied the mixed logistic regression to assess the trend of observed HOT use with increasing provincial GDP per capita.

To determine the association between observed HOT use and air pollution, we applied mixed logistic regression using HOT as a dependent variable and particulate emission matters as an independent variable. The data on particulate emission matter were obtained from National Bureau of Statistics of China [25]. Furthermore, a linear regression model was applied to determine the association between provincial GDP per capital and particulate emission matter.

We did not impute missing data. Data management and analysis were conducted using SAS, version 9.4 (SAS Institute, Cary, North Carolina) and the *R* package ggplot2 (version 3.6.1). Our two-tailed significance level was set at *P* < 0.05.

## Results

### Demographic data and characteristics of HOT

A total of 10,823 very preterm or very low birthweight infants were admitted to 57 CHNN member hospitals in 2019 and were assessed for eligibility. Ultimately, 1768 of the 10,823 infants who were diagnosed with BPD and discharged from 56 hospitals met the inclusion criteria. Overall, 474 (26.8%) and 1294 (73.2%) infants were discharged to home with or without HOT, respectively (Fig. 1).

Table 1 indicates the characteristics of HOT in preterm infants with bronchopulmonary dysplasia. The proportions of different percentiles of birthweight, doses of surfactant use, severe NEC (≥ stage II), NO use, accumulative days of stay in the NICU, and invasive ventilation support were significantly different between the groups. Among infants with HOT, the average length of stay in NICU in the NEC group (73.5 days) was longer than that of the non-NEC group (58.5 days). Maternal factors that included delivery mode and course of antenatal steroids (AS) were similar between the two groups. There was no significant difference in the proportion of HOT use with respect to sepsis, PDA ligation, inhaled or systemic steroid use, or delivery room resuscitation. Duration of breast milk feeding, days of non-invasive ventilation, and weight gain in the NICU did not show any significant difference between the two groups.

**Table 1:** Characteristics of home oxygen therapy (HOT) in preterm infants with bronchopulmonary dysplasia

Factors	Level	*n*	Discharge without HOT (*N* = 1294)	Discharge with HOT (*N* = 474)	*P* value
Maternal factors					
Delivery type, *n *(%)	Vaginal delivery	813	586/813 (72.1%)	227/813 (27.9%)	0.32
	Cesarean section	941	698/941 (74.2%)	243/941 (25.8%)	
AS, *n* (%)	No	368	259/368 (70.4%)	109/368 (29.6%)	0.71
	Yes	1185	846/1185 (71.4%)	339/1185 (28.6%)	
Full course of AS, *n* (%)^a^	No	508	357/508 (70.3%)	151/508 (29.7%)	0.46
	Yes	677	489/677 (72.2%)	188/677 (27.8%)	
Neonatal factors					
Birth weight, *n* (%)	< 500 g	1	0/1 (0.0%)	1/1 (100.0%)	0.001
	500-749 g	55	42/55 (76.4%)	13/55 (23.6%)	
	750-999 g	394	313/394 (79.4%)	81/394 (20.6%)	
	1000-1249 g	605	451/605 (74.5%)	154/605 (25.5%)	
	1250-1499 g	425	296/425 (69.6%)	129/425 (30.4%)	
	> 1499 g	288	192/288 (66.7%)	96/288 (33.3%)	
Gestational age, wk, *n* (%)	< 26 wk	87	59/87 (67.8%)	28/87 (32.2%)	0.49
	26–28 wk	694	512/694 (73.8%)	182/694 (26.2%)	
	29–31 wk	987	723/987 (73.3%)	264/987 (26.7%)	
Percentile of birth weight, *n* (%)^b^	SGA	177	148/177 (83.6%)	29/177 (16.4%)	0.004
	AGA	1559	1123/1559 (72.0%)	436/1559 (28.0%)	
	LGA	28	20/28 (71.4%)	8/28 (28.6%)	
Sepsis, *n* (%)	No	1516	1117/1516 (73.7%)	399/1516 (26.3%)	0.25
	Yes	252	177/252 (70.2%)	75/252 (29.8%)	
NICU length of stay, d, mean (SD)		1768	66.88 (23.96)	58.93 (29.63)	< 0.001
Duration of breast milking per 100 NICU stay, median (IQR)		1768	65 (0,90)	70 (18,91)	0.052
Weight gain in NICU, g/kg/d, median (IQR)		1762	11 (9.3,12.6)	11 (9.1,12.7)	0.79
Doses of surfactant use, *n* (%)	0	755	569/755 (75.4%)	186/755 (24.6%)	0.008
	1	791	581/791 (73.5%)	210/791 (26.5%)	
	2	171	115/171 (67.3%)	56/171 (32.7%)	
	≥ 3	51	29/51 (56.9%)	22/51 (43.1%)	
PDA ligation, *n* (%)	No	1714	1253/1714 (73.1%)	461/1714 (26.9%)	0.72
	Yes	37	28/37 (75.7%)	9/37 (24.3%)	
NEC stage ≥ Stage II, *n* (%)	No	1657	1198/1657 (72.3%)	459/1657 (27.7%)	0.001
	Yes	111	96/111 (86.5%)	15/111 (13.5%)	
NO use, *n* (%)	No	1739	1279/1739 (73.5%)	460/1739 (26.5%)	0.009
	Yes	29	15/29 (51.7%)	14/29 (48.3%)	
Inhaled steroids use, *n* (%)	No	1409	1020/1409 (72.4%)	389/1409 (27.6%)	0.13
	Yes	359	274/359 (76.3%)	85/359 (23.7%)	
Systemic steroids use, *n* (%)	No	1248	924/1248 (74.0%)	324/1248 (26.0%)	0.21
	Yes	520	370/520 (71.2%)	150/520 (28.8%)	
Delivery room resuscitation, *n* (%)	None	568	432/568 (76.1%)	136/568 (23.9%)	0.16
	Bag & mask	254	181/254 (71.3%)	73/254 (28.7%)	
	CPAP	126	90/126 (71.4%)	36/126 (28.6%)	
	Intubation	561	387/561 (69.0%)	174/561 (31.0%)	
	Compression and epinephrine	106	79/106 (74.5%)	27/106(25.5%)	
Post-delivery room respiratory support, d, median (IQR)^c^	Invasive ventilation	1155	8 (4, 17)	8.5 (4, 21)	0.07
	Non-invasive ventilation	1672	22 (11, 35)	20.5 (10.5, 32)	0.17

*AS* antenatal steroids, *SGA* small for gestational age, *AGA* appropriate for gestational age, *LGA* large for gestational age, *NICU* neonatal intensive care unit, *IQR* interquartile range, *PDA* patent ductus arteriosus, *NEC* necrotizing enterocolitis, *NO* nitric oxide, *CPAP* continuous positive airway pressure. ^a^Calculation within infants who received AS. ^b^Based on Zhu L., et al^18^. ^c^Calculation Based on population who did specific respiratory support

### Risk factors for HOT use

Multivariable analysis was used to identify predictive factors for HOT (Table 2). We observed significantly higher odds of HOT use in infants born less than 26 weeks of gestational age [adjusted odds ratio (aOR)], 3.17; 95% confidence interval [(CI) 1.60–6.28, *P* = 0.03], infants born between 26 and 28 weeks of gestational age [aOR, 1.55 (1.17–2.05, *P* = 0.002)], and those infants requiring ≥ 3 doses of surfactant [aOR 2.22 (1.14–4.32), *P* = 0.03]. In addition, with a 7-day increase in hospital stay and invasive ventilation, the adjusted odds ratio of HOT was [0.74 (0.68–0.82) and 1.24 (1.13–1.35)], respectively.

**Table 2:** Multivariate analysis of risk factors for home oxygen therapy (HOT)

Factors	Level	Adjusted OR	*P* value
Gestational age, wk	29–31 wk	Ref	
	26–28 wk	1.55 (1.17, 2.05)	0.002
	< 26 wk	3.17 (1.60, 6.28)	0.03
Percentile of birth weight	AGA	Ref	
	SGA	0.64 (0.35, 1.15)	0.13
	LGA	0.87 (0.26, 2.91)	0.83
NICU length of stay, 7-unit increase^a^		0.74 (0.68, 0.82)	< 0.001
Duration of breast milking per 100 NICU stay, 1-unit increase		1.02 (0.64, 1.65)	0.92
Doses of surfactant use	0	Ref	
	1	1.24 (0.85, 1.81)	0.27
	2	1.31 (0.77, 2.25)	0.32
	≥ 3	2.22 (1.14, 4.32)	0.03
NO use	No	Ref	
	Yes	1.39 (0.63, 3.08)	0.42
NEC ≥ stage II	No	Ref	
	Yes	0.44 (0.16, 1.18)	0.16
Invasive ventilation, 7-unit increase^a^		1.24 (1.13, 1.35)	< 0.001

*OR* odds ratio, *Ref* reference, *SGA* small for gestational age, *AGA* appropriate for gestational age, *LGA* large for gestational age, *NICU* neonatal intensive care unit, *NO* nitric oxide, *NEC* necrotizing enterocolitis. ^a^The change trend of OR value for every 7 day of continuous variable increase

### Institutional variations in HOT use

**Fig. 2:**
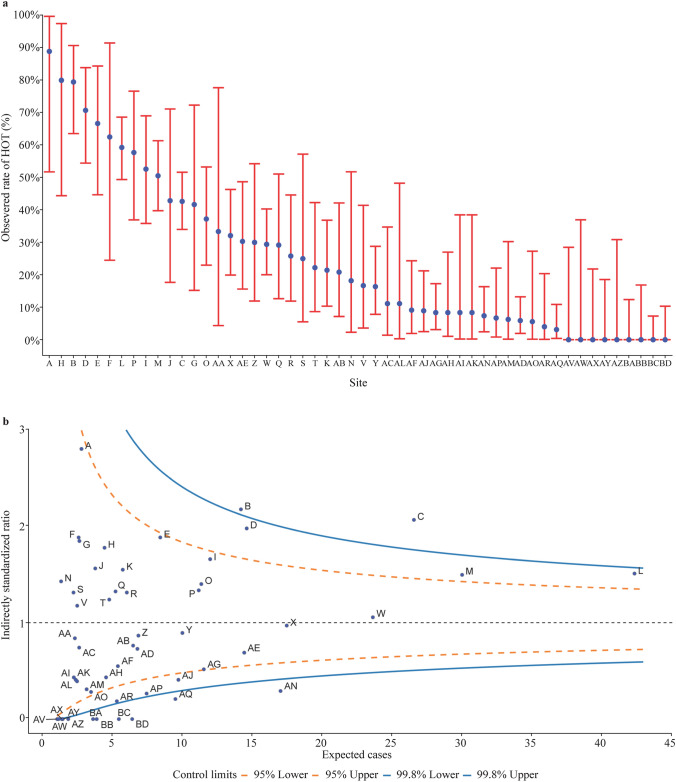
Institutional variation of home oxygen therapy. **a** The proportion of home oxygen therapy in participating NICUs of the Chinese Neonatal Network; **b** The “Funnel” plot of home oxygen therapy

Institutional variation was analyzed among 52 NICUs that discharged ≥ 5 infants with BPD in 2019. Fig. 2a shows significant institutional variations in HOT, with observed proportions in different NICUs ranging from 0 to 89%.

The expected proportion of HOT in each NICU was calculated after adjustment of relevant factors (Table 2). Fig. 2b illustrates variation in the observed and expected proportions of HOT among the 52 CHNN centers that discharged at least five patients with BPD during the study period. Five and 14 hospitals exceeded + 95% and − 95% of the control limit, respectively, suggesting that these hospitals had significantly higher and lower use of HOT at discharge compared to that of the total study population (reference population). Collectively, 36.5% (19/52) of hospitals implemented greater or less HOT than the expected number.

### Relationship between use of HOT and provincial economic level and air pollution

**Fig. 3:**
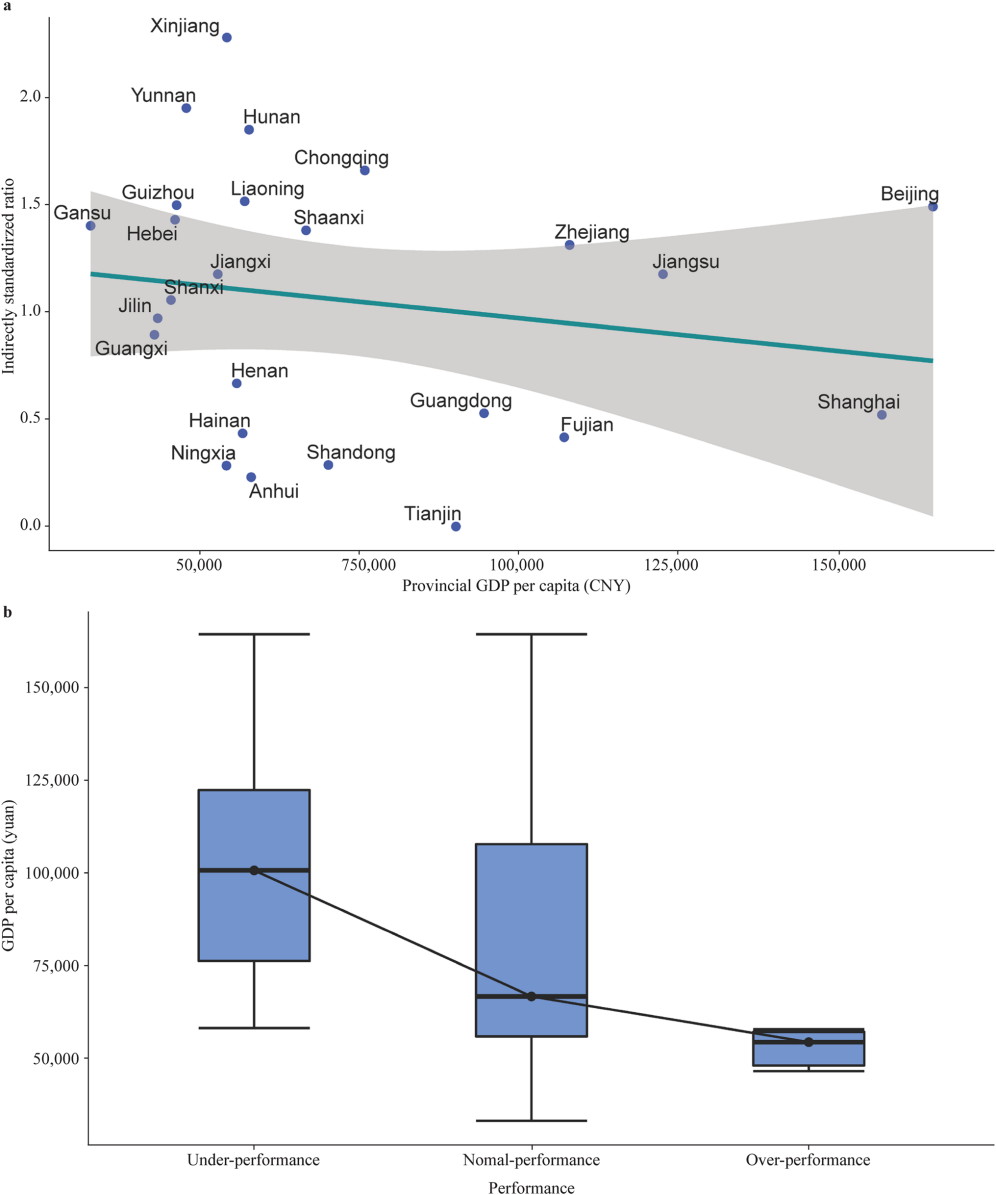
Relationship between institutional home oxygen therapy and provincial economic level. **a** Correlation between indirectly standardized ratio of home oxygen therapy and Per capita GDP of provinces; **b** The tendency of GDP per capita among different performance groups of home oxygen therapy

Fig. 3a shows a downward trend in the indirectly standardized ratio of HOT with increasing provincial GDP per capita (*P* = 0.45). 71.4% (5/7) of provinces with GDP per capita ≤ 50,000 CNY/year, 50% (5/10) provinces with GDP per capita between 50,000 and 75,000 CNY/year and 50% (4/8) provinces with GDP per capita ≥ 75,000 CNY/year showed greater observed proportions of HOT use than expected proportion of HOT use. There was a significantly decreased percentage of HOT use (*P* = 0.04) with increase of the provincial GDP per capital (Supplemental Fig. 1). As shown in the supplemental Fig. 2, the use of HOT increased as increased provincial annual emission of particulate matter (*P* = 0.26). In addition, supplemental Fig. 3 showed that the provincial annual emission of particulate matters was negatively associated with provincial GDP per capital (*P* < 0.01).

Five of 52 hospitals exhibited observed proportions of HOT use that were significantly greater than expected (i.e., the over-performing group), 14 hospitals had observed proportions of HOT that were significantly less than expected (the underperforming group), and 33 hospitals manifested appropriate proportions of HOT use (the normal-performing group). Figure 3b shows that GDP per capita was highest in the underperforming group and was lowest in the over-performing group (*P* < 0.01).

## Discussion

The purpose of this study was to characterize the use of HOT in preterm infants with BPD in China based on the Chinese Neonatal Network data, so as to generate beneficial information in the design of interventions and the formulation of appropriate HOT policy.

In our cohort, we identified clinical risk factors associated with higher use of HOT for preterm infants with BPD that were similar to those of previous studies—including small for gestational age at birth, more doses of surfactant needed, shorter NICU stays, and a longer duration on invasive ventilation.

Table 1 shows that low incidence of severe NEC in babies discharged with HOT was noticed. Actually, the percentage of severe NEC and death in infants who met the exclusion criteria was higher than that of the study population, suggesting that low incidence of NEC on HOT may be attributed to early death caused by NEC. Among infants with HOT, the length of stay in NICU in the NEC group (73.5 days) was longer than that of the non-NEC group (58.5 days), which indicated that patients with severe NEC had a chance to receive longer respiratory support in the hospital. As a result, the longer length of stay allowed time for recovery from BPD, and thus lower HOT use was observed.

Although previous studies indicated that PDA ligation and SGA were related to a higher proportion of HOT use [11, 12], in our cohort, these indices were not significantly correlated with higher odds of HOT use after multivariable analysis. Among our patients with HOT, only nine patients underwent PDA ligation and 29 patients were born SGA. Our small sample size may have led to bias in our results; so future studies should require the enrollment of a larger number of infants to explore the potential risk factors involved in producing a higher rate of HOT use in China.

HOT has been indicated for some preterm infants with severe bronchopulmonary dysplasia (BPD) upon discharge since the 1970s [6], and studies have shown that HOT engenders potential familial and economic benefits. For example, HOT can reduce the average length of a hospital stay by 1.5 weeks [11], reduce economic burden [6], prevent the impact of chronic hypoxemia, enhance the overall quality of life in children, and improve the impact of the family environment on family members of infants without increased risk of readmission [27].

There is currently no worldwide consensus for HOT use in preterm infants with BPD. The recent guidelines from the Thoracic Societies in Britain, Australia, New Zealand, and the United States differ markedly on some issues. For example, the delineation of relatively stable newborns who can be discharged with home oxygen remains controversial [28]. According to a survey, there was no national guideline in Germany, and this resulted in a wide range of SpO2 cutoff values indicated for HOT in different facilities that ranged from 80% to 94% [29]. The reported rate of HOT use in other countries varied from 13.2 to 65.2% among BPD preterm infants, with wide variations in institutional proportions from 7% to 95% [8, 11, 12]. In our cohort, HOT was used in 26.8% of preterm Chinese infants with BPD, which was lower than the proportions of HOT use for infants with BPD in the United States and United Kingdom, but higher than those for South Korea, New Zealand, and Australia [8, 9].

With respect to the factors that contribute to the wide variation in institutional proportions of HOT, physician as well as parent preferences regarding HOT for infants with BPD might play a significant role [30]. In our cohort there were more than 1/3 of hospitals with an observed use of HOT that was significantly greater or less than expected, with a proportion of HOT that varied between 0 and 89% among infants with BPD in different institutes. This result is similar to the wide institutional variation noted in other reports [12]. Future study is needed to further address the aforementioned contributions to the use of HOT in China.

In addition, the distribution of social resources occupies an important role in HOT. The premature infants who were discharged with HOT needed more support with respect to community health care. There should be special nursing teams pursuing long-term follow-up, professional nurses for regular sleep research, and medical and health professionals for additional home visits to comprehensively evaluate the family environment. When parents suspect that their baby has dyspnea, pediatric community medical staff can then evaluate the neonates and provide general respiratory support [31].

Regional pediatric community health services vary in China. A survey in 2014 showed that the proportion of children under the age of 15 who used community services was only 2.4%, that the training of community health-institution doctors in children's health care was limited, and that most community-service institutions did not provide inpatient services [32].

Distinct from our initial assumption, we have now recognized that the regional proportion of HOT use was negatively correlated with the level of provincial economic development. Spooner et al. identified key differences that exist across patient and hospital characteristics with respect to discharged against medical advice and found that lack of health insurance (OR 3.78; 95% CI 3.62–3.94) was one of the major predictors [33]. We speculated that the negative correlation in our cohort might be related to the fact that parents in cities with a higher economic level might possess satisfactory insurance coverage or financial support for hospitalization expenses, and thus prefer to stay in the hospital for a longer period of time. In contrast, parents in cities exhibiting a lower economic level might not have sufficient insurance coverage or financial support for their hospital expenses and prefer early discharge with HOT. The causes underlying the relationship between institutional use of HOT and GDP per capita also warrant additional clarification in future studies. Given this intriguing phenomenon, efforts should be made to improve local community medical-service systems to provide sufficient support for preterm infants discharged with HOT. In addition, local—but not national—HOT guidelines are needed in countries that exhibit a wide variation in HOT use and in those with disparate regional economic levels, such as China.

Supplemental Figs. 2 and 3 suggested that more emission of particulate matter was associated with both lower GDP per capital and more HOT use. The air pollution may slow infants’ respiratory recovery at home, which may be related to increase of oxygen use at home. However, this relationship may be biased due to potential confounders, such as smoking status of parents and air quality of home. In future, we will further investigate the association of HOT use with the air pollution due to lack of data on air quality at home.

This is the first study to characterize the use of HOT in preterm infants with BPD in China. The strengths of this study included its large sample size and our valid and reliable data collection system. Data were collected prospectively from 57 tertiary centers located in 27 regions of China and adequately represented the characteristics of HOT use in China, thus avoiding bias to an extent.

However, the findings from our study possess several limitations that require consideration when interpreting the data. First, the survival rate for premature infants who were very small for gestational age or showed very low weight at birth was low. Investigators therefore need to enroll more infants in future to explore the actual use of HOT in such groups of Chinese infants. Second, because treatment of BPD varied at different centers and there are no extant guidelines for HOT, this might have also resulted in bias. Finally, long-term follow-up is required to compare the prognosis of infants discharged with HOT to those without HOT.

In conclusion, we demonstrated that the use of HOT varied across China and was negatively correlated with economic development at the provincial level. These findings suggest that local HOT guidelines are needed in countries such as China that display a wide variation in HOT use and different regional economic levels.

## Supplementary Information

Below is the link to the electronic supplementary material.

Supplementary file1 (PDF 375 kb)

## Data Availability

The original contributions presented in the study are included in the article/supplementary material, further inquiries can be directed to the corresponding authors.
