# Supplementary material for: Characteristics of home oxygen therapy for preterm infants with bronchopulmonary dysplasia in China: results of a multicenter cohort study
Source: World J Pediatr. 2022 Aug 11;19(6):557–67. doi: 10.1007/s12519-022-00591-9 (PMC10198895; doi:10.1007/s12519-022-00591-9)
Supplement: Supplementary file 1 — Supplementary file1 (PDF 375 kb) [file 12519_2022_591_MOESM1_ESM.pdf]

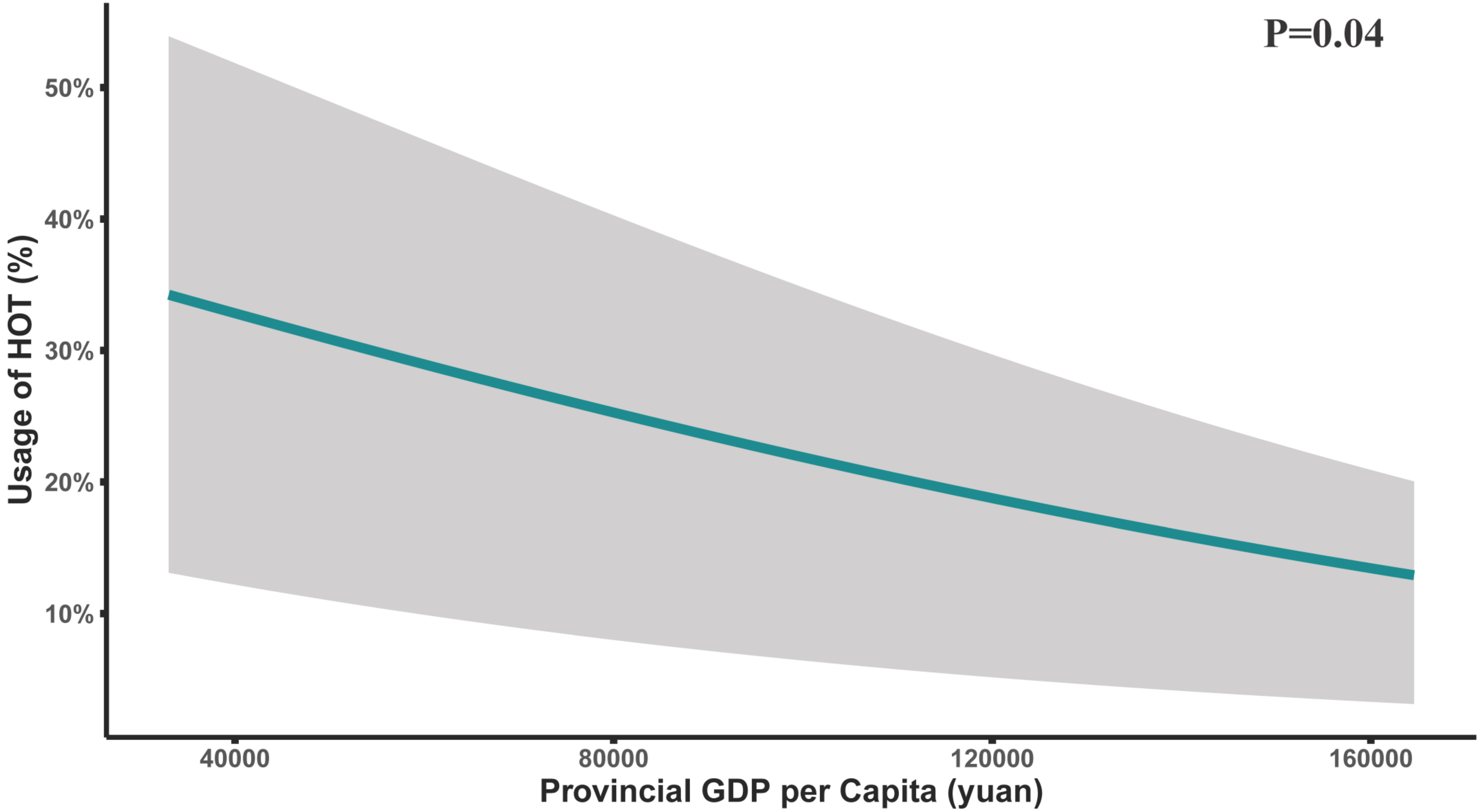

Supplemental Fig.1. Relationship between usage of home oxygen therapy and provincial economic level using the mixed logistic regression model.

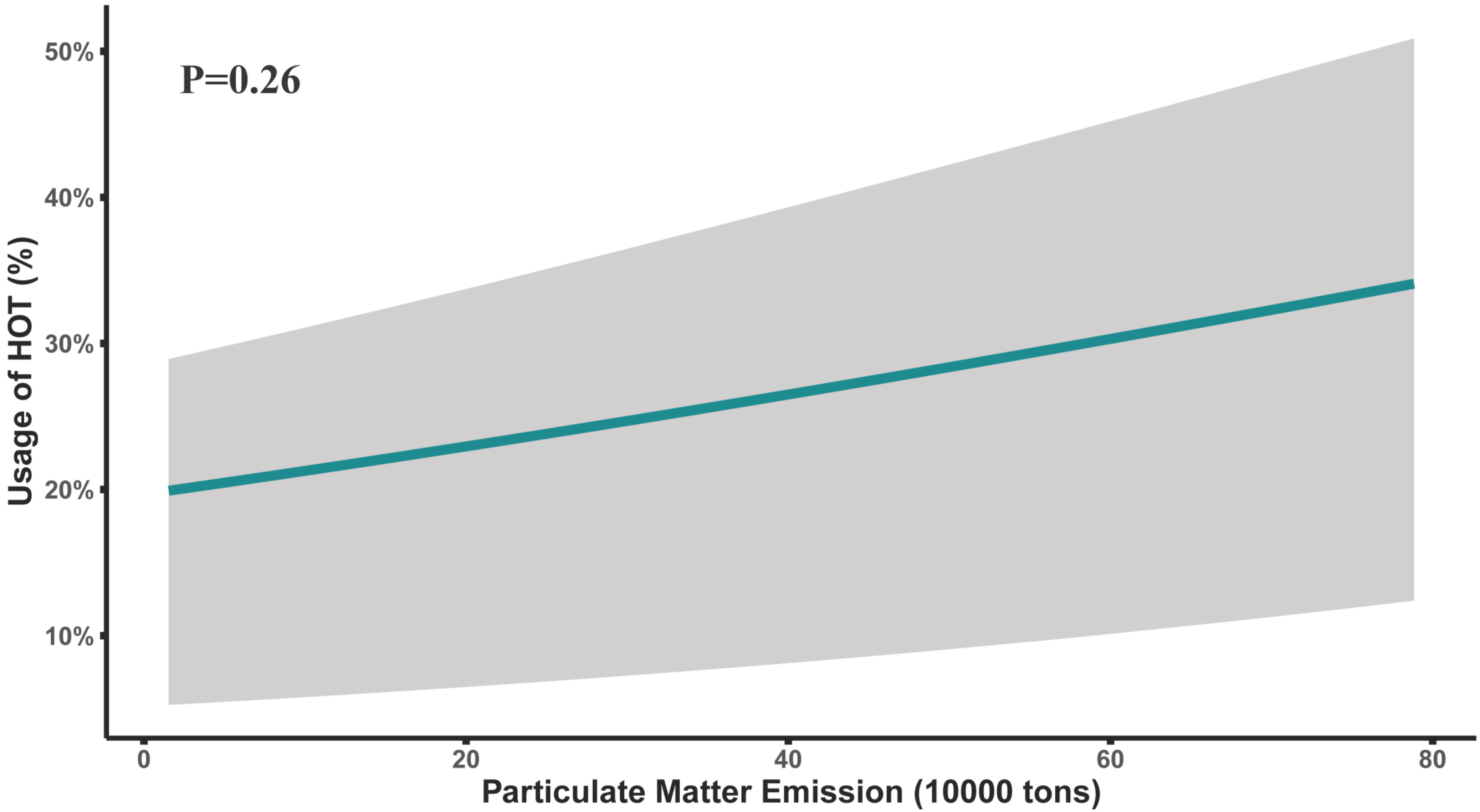

Supplemental Fig.2. Influence of provincial annual emission of particulate matter on usage of home oxygen therapy

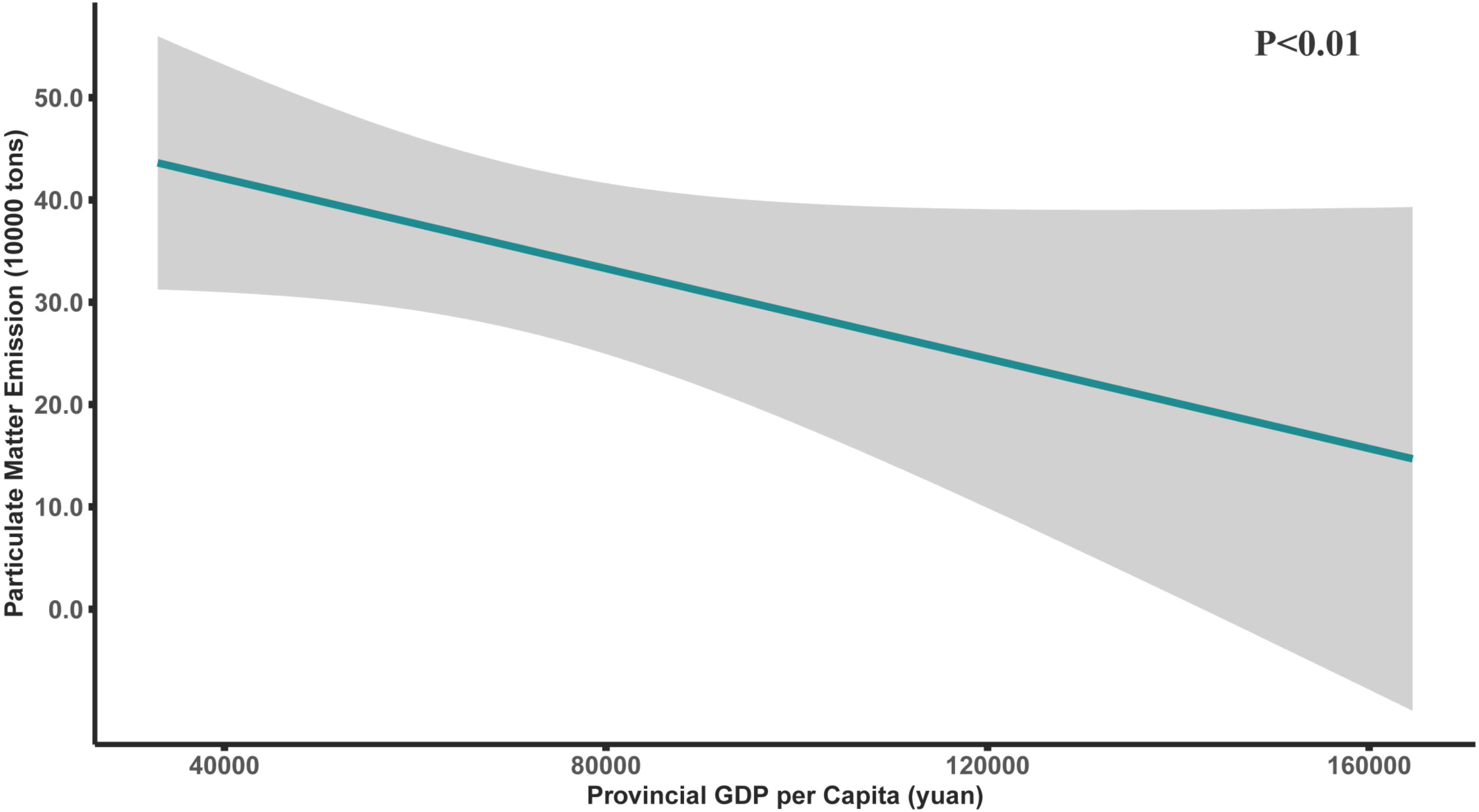

Supplemental Fig.3. Association of provincial economic level with provincial annual emission of particulate matter.
